# Second report of registry of the International Society of Uterus Transplantation (ISUTx): international activities 2000–2024

**DOI:** 10.1093/humrep/deag017

**Published:** 2026-02-17

**Authors:** Mats Brännström, Catherine Racowsky, Johanna Wiik, Stefan G Tullius, Rebecca Deans, Paige M Porrett, Wellington Andraus, Tan Hak Koon, Francisco Carmona, Randa Akouri, Paolo Scollo, Smit Solanki, Padmapriya Vivek, J Richard Smith, Shailesh P Puntambekar, Sung Eun Kim, Ömer Özkan, Jean-Marc Ayoubi, Jiri Fronek, Sara Y Brucker

**Affiliations:** Department of Obstetrics and Gynecology, Institute of Clinical Sciences, Sahlgrenska Academy, University of Gothenburg, Göteborg, Sweden; Department of Obstetrics, Gynecology and Reproductive Biology, Harvard Medical School, Boston, MA, USA; Department of Obstetrics and Gynecology, Institute of Clinical Sciences, Sahlgrenska Academy, University of Gothenburg, Göteborg, Sweden; Division of Transplant Surgery, Brigham and Women’s Hospital, Harvard Medical School, Boston, MA, USA; Royal Hospital for Women, University of New South Wales, Sydney, NSW, Australia; Department of Surgery, University of Alabama at Birmingham, Birmingham, AL, USA; Digestive Organs Transplant Division, Gastroenterology Department, University of São Paulo School of Medicine FM-USP, São Paulo, Brazil; Department of Obstetrics and Gynecology, Singapore General Hospital, Singapore, Singapore; Institute Clinic of Gynecology, Obstetrics and Neonatology, Hospital Clinic de Barcelona, Institut dÍnvestigacions Biomèdiques August Pi i Sunyer, Universitat de Barcelona, Barcelona, Spain; Department of Obstetrics and Gynecology, Institute of Clinical Sciences, Sahlgrenska Academy, University of Gothenburg, Göteborg, Sweden; Bellevue Medical Center, St. Joseph University, Beirut, Lebanon; Division of Gynecology and Obstetrics, Maternal and Child Department, Cannizzaro Hospital, Catania, Italy; Department of Obstetrics and Gynecology, Institute of Kidney Diseases and Research Center, Dr. H.L. Trivedi Institute of Transplantation Sciences, Ahmedabad, Gujarat, India; Department of Obstetrics and Gynecology, Gleneagles Global Health City, Chennai, Tamil Nadu, India; West London Gynaecological Cancer Centre, Hammersmith Hospital, Imperial College, London, UK; Department of Surgery, Galaxy Care Multi-Speciality Hospital, Pune, Maharashtra, India; Department of Obstetrics and Gynecology, Samsung Medical Center, Sungkyunkwan University School of Medicine, Seoul, South Korea; Department of Plastic and Reconstructive Surgery, Akdeniz University Faculty of Medicine, Antalya, Turkey; Department of Gynecology, Foch Hospital, Suresnes, France; Department of Transplantation Surgery, Institute for Clinical and Experimental Medicine, Prague, Czech Republic; Department of Women’s Health, University Hospital of Obstetrics and Gynecology Tubingen, Eberhard Karls University, Tubingen, Germany

**Keywords:** uterus, infertility, transplantation, live birth, rejection, assisted reproduction, donor, recipient, surgery, immunosuppression

## Abstract

**STUDY QUESTION:**

What have been the activities, characteristics, and outcomes of uterus transplantation (UTx) performed worldwide from 2000 through 2024?

**SUMMARY ANSWER:**

In 91 UTx cases, 67 involved live donors and 80 of the recipients had Mayer-Rokitansky-Küster-Hauser syndrome, with 12-month graft survival of 74%, enabling pregnancy attempts that yielded 44 healthy singleton deliveries with a live birth rate per embryo transfer of 30.3%.

**WHAT IS KNOWN ALREADY:**

UTx is the only treatment for women with absolute uterine factor infertility who wish to carry a pregnancy. According to a comprehensive report including data up to 2020 on 45 UTx cases, 19 live births occurred (35.8% per embryo transfer) at a mean of 35.3 weeks gestation.

**STUDY DESIGN, SIZE, DURATION:**

Data were extracted from the web-based registry of the International Society of Uterus Transplantation (ISUTx). This registry captures information on donor and recipient characteristics, transplantation procedures, postoperative complications, immunosuppression, complications including rejections, and reproductive outcome. Analyses were undertaken of the 91 transplants performed between 6 April 2000 and 31 December 2024, that were recorded in the registry.

**PARTICIPANTS/MATERIALS, SETTING, METHODS:**

Twenty-four medical centers in five continents registered their uterus transplants by entering data from the day of transplantation until 3 months after graft removal. The following variables were assessed: the demographic and laboratory characteristics of donors and recipients, the source of graft (live versus deceased donor), surgical specifics including technique, duration, ischemic times, and post-op complications, immunosuppression, rejection data, pregnancy with live birth(s), and hysterectomy.

**MAIN RESULTS AND THE ROLE OF CHANCE:**

In 91 uterus transplantations (67 from live donors, 24 from deceased donors), the overall surgical success rate, defined as graft viability by 12 months, was 75%. Most recipients (88%) had Mayer-Rokitansky-Küster-Hauser syndrome, with mothers being the most frequent (64%) live donors. Live donor hysterectomies were performed by laparotomy (54%), robotics (28%), or laparoscopy (18%). Total ischemic time was shorter in live- versus deceased-donor UTx procedures. Rejection episodes that were treated with escalations of immunosuppression were more frequent during months 1–5 (44%) than during months 6–10 (28%) post-UTx. Graft survival during the first 12 months was superior when grafts from premenopausal donors were used as compared to from postmenopausal donors. Forty-four singleton live births (mean gestational length of 34.5 weeks), including eight second births, were reported, with a live birth rate per embryo transfer of 30%. Preeclampsia was the most common pregnancy complication, occurring in 23% of live-birth pregnancies. Major postnatal complications occurred in 11 infants, 9 with respiratory distress syndrome; no major malformations were observed.

**LIMITATIONS, REASONS FOR CAUTION:**

Data in the registry are self-reported and not subjected to validation. Although the ISUTx registry represents the most comprehensive quality registry of UTx activity in the world, cases from at least four centers are excluded as they were not entered into the registry. Birth outcomes from some registry cases are as yet unknown as these ongoing cases have not yet reached the endpoint of hysterectomy.

**WIDER IMPLICATIONS OF THE FINDINGS:**

This study presents the most comprehensive analysis to date of UTx, the only fertility treatment for absolute uterine factor infertility. The registry serves as the prime source for quality assessment and process improvement in UTx.

**STUDY FUNDING/COMPETING INTEREST(S):**

The establishment and operation of the registry were funded by the Swedish Research Council (2024-03487 to M.B.) and Jane and Dan Olsson Foundation (2024-11 to M.B.). There was no competing interest.

**TRIAL REGISTRATION NUMBER:**

Not applicable.

## Introduction

Uterus transplantation (UTx) has developed from an experimental procedure to an established clinical approach, representing the only treatment option for women with absolute uterine factor infertility (AUFI). This infertility condition is due to a lack of a uterus because of congenital absence or surgical removal, or the presence of a uterus that is unable to sustain a pregnancy for a duration that permits neonatal viability (Hur *et al.*, 2019). Following extensive and systematic animal research spanning over a decade (Brännström *et al.*, 2012), the first live birth following UTx was achieved in Sweden in 2014 (Brännström *et al.*, 2015), establishing the clinical feasibility of this novel fertility treatment.

Over the last decade, the field has advanced substantially, with the initiation of structured clinical trials at several sites, multidisciplinary research, and increased international collaborations. Rapid growth of the field is further illustrated by the emergence of several clinical programs during recent years, which have performed UTx procedures outside of the designation of formal clinical trials. In 2017, the International Society of Uterus Transplantation (ISUTx) was established to facilitate scientific exchange, promote best practices, and maintain a global registry of UTx procedures (Tummers *et al.*, 2019).

The ISUTx registry was launched in 2020 and serves as the primary platform for tracking activity, donor and recipient characteristics, surgical data, immunosuppression protocols, complications, rejection episodes, and reproductive outcomes. The first registry report, covering 45 UTx procedures up until 2020, highlighted both the progress and challenges of the field (Brännström *et al.*, 2023a). That report documented 19 live births, all following IVF, with a live-birth rate per embryo transfer (ET) of 35.8% and a mean gestational age at delivery of 35.3 weeks. However, despite promising reproductive outcomes, the UTx procedure remains complex, with substantial variation in surgical techniques among centers and a notable incidence of postoperative complications for both recipients and live donors (LDs).

This second UTx registry report includes analyses of all data submitted up to December 31, 2024, providing a robust collection of global activities that will serve as the key framework moving our field forward. Here, we present updated data from previously reported cases (Brännström *et al*., 2023a) as well as the inclusion of data from new cases performed at original and new centers. This report illustrates changing trends in donor and recipient selection, as well as surgical approach, immunological management, and reproductive outcomes. As UTx continues to evolve from an experimental trial to clinical treatment, comprehensive registry data remain essential for guiding clinical practice, refining protocols, and shaping the future of UTx.

## Materials and methods

### Participating centers

Twenty-four centers contributed data on a total of 91 UTx procedures performed between April 2000 and December 2024. The participating centers (with the year of their first case and number of cases in parentheses) were chronologically: King Fahad Hospital and Research Center, Jeddah, Saudi Arabia (2000; n = 1); Akdeniz University Hospital, Antalya, Turkey (2011; n = 2); Sahlgrenska University Hospital, Gothenburg, Sweden (2012; n = 20); The First Affiliated Hospital of Air Force Medical University Xijing Hospital, Xi′an, China (2015; n = 2); Hospital das Clinicas/Sao Paulo University, Sao Paulo, Brazil (2016; n = 3); Institute for Clinical and Experimental Medicine, Prague, Czech Republic (2016; n = 10); Tübingen University Hospital, Tübingen, Germany (2016; n = 7); Zhujiang Hospital, Southern Medical University, Guangzhou, China (2017; n = 1); University Children Hospital Tirsova, Belgrade, Serbia (2017; n = 1); Galaxy Care Hospital, Pune, India (2017; n = 9); Bellevue MC/St. Joseph Univ, Beirut, Lebanon (2018; n = 3); Universitair Ziekenhuis (UZ) Ghent, Ghent, Belgium (2018; n = 1); Star Médica Hospital, Chihuahua, Mexico (2019; n = 1); Hospital Foch, Suresnes/Paris, France (2019; n = 3); Barretos Cancer Hospital, Sao Paulo, Brazil (2019; n = 1); Cannizzaro Hospital, Catania, Italy (2020; n = 3); Hospital Clinic de Barcelona, Barcelona, Spain (2020; n = 3); UAB Medicine, University of Alabama at Birmingham, Birmingham, ALA, USA (2022; n = 5); Samsung Medical Center, Sungkyunkwan University School of Medicine, Seoul, South Korea (2022; n = 3); Institute of Kidney Diseases and Research Center, Gujarat, India (2022; n = 2); Gleneagles Global Health City, Chennai, India (2022; n = 2); The Royal Hospital for Women, Sydney, Australia (2023; n = 3); Hammersmith Hospital, Imperial College London, London, United Kingdom (2023; n = 4); SingHealth Duke-NUS, Academic Medical Center Singapore, Singapore (2023; n = 1). We are aware of four centers worldwide (three in the USA [Dallas, Cleveland, Philadelphia] and one in Australia [Sydney]) that have not submitted their data into the registry and so are not included in this report.

### ISUTx registry

The ISUTx registry is a web-based platform designed to collect data prospectively. Data included in the registry are self-reported and not subjected to validation. The registry captures detailed information on each UTx procedure, continuing through to 3 months after a transplant hysterectomy, whether performed following live birth(s), after multiple unsuccessful pregnancy attempts, or due to graft failure. The data are reported anonymously. Therefore, donor and recipient ages are recorded only as completed whole years on the day of UTx.

Data fields are provided, covering donor and recipient characteristics, surgical details (including donor hysterectomy and transplantation), immunosuppression (IS), episodes of rejection, pregnancies, details on assisted reproduction, live births, and transplant hysterectomies. The comprehensive list of data variables and structured multiple-choice fields included in the registry were described in detail in the first registry report (Brännström *et al.*, 2023a).

Postoperative complications occurring within 90 days after surgery are classified using the Clavien-Dindo (CD) system (Dindo *et al.*, 2004). Grade I encompasses minor deviations from the expected postoperative course, and Grade II includes complications necessitating non-standard therapies such as antibiotics, blood transfusions, or total parenteral nutrition. Grade III refers to complications that require radiologic, endoscopic, or surgical intervention—distinguished as IIIa (without general anesthesia) or IIIb (with general anesthesia). Grade IV includes life-threatening complications. In the registry, complications ≥ CD I, occurring within 90 days after surgery, are registered both for the LD and the recipient. However, details on the specific types of complications within each CD grade are not entered.

**Figure 1.**
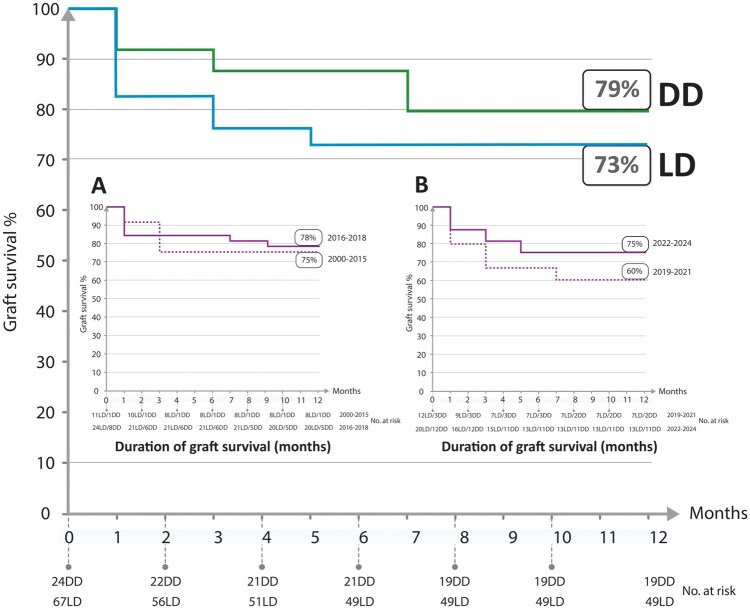
**Graft survival during the initial 12 months after uterus transplantation for live donor (LD) and deceased donor (DD) transplantation procedures.** To simplify visualization, all graft losses that occurred between even months (e.g. between months 0–2, 2–4, etc.) are indicated at the intermediate odd month (1, 3, 5, 7, 9, or 11). Insert A and B show the total (LD+DD) graft survival during four different time periods.

Graft survival at 12 months was defined as a graft remaining in situ in the recipient one year after UTx. Nine of the 91 procedures had been performed within the last 12 months, and thereby these had not included the full time for analysis. One of the nine grafts of the year 2024 had been removed, within the first month, and is included as a graft loss in the graph of graft survival of 12 months (Fig. 1). The eight ongoing grafts from 2024 had observation times of 10 months (n = 1), 9 months (n = 3), 6 months (n = 1), 4 months (n = 1), and 3 months(n = 2). Possible listed reasons for graft removal included thrombosis, rejection, intrauterine infection, cervical premalignant condition, uterine premalignant condition, malignancy, patient’s request, recurrent miscarriage or implantation failure, side-effects of immunosuppression, uterine hypoperfusion, irreversible endometrial damage, post-transplant lymphoproliferative disorder (PTLD), and unspecified. Of note, reasons for graft removal were defined by the registry, and only one main reason for graft removal could be selected by the center entering the data.

Rejections were entered as mild, moderate, or severe. Although most centers have been advised to grade rejections according to the original grading system for monitoring rejection after UTx (Mölne *et al.*, 2017), it is possible that some did not strictly adhere to the Mölne system. Thus, rejection grading may vary slightly between centers. Data on serology of cytomegalovirus (CMV), Epstein-Barr virus (EBV), and Toxoplasma were collected according to the platform for analyses, used at each center. Data on high-risk Human Papillomavirus (hrHPV) were analyzed according to the criteria of each center’s chosen platform, including its classification of specific HPV subtypes as high-risk.

Determination of live birth rate was restricted to UTx procedures performed before July 2023 to allow sufficient time for at least three ETs and a first pregnancy with live birth to be recorded for each recipient by 31 December 2024.

### Data presentation and statistics

Analyses were performed using SPSS software (version 30.0 IBM, https://www.ibm.com/analytics/spss-statistics-software). Data are presented as percentages or numeric values, along with the mean/median and standard deviation (SD)/ranges for variables. Background characteristics of live and deceased donors were compared by chi-square or Student′s *t*-test as appropriate. A *P*-value < 0.05 was selected as the threshold of statistical significance. Associations between graft survival for 12 months and age of donor, type of donor, menopausal status of donor, total ischemic time, rewarming ischemic time, surgical time of LD, and surgical time of recipient were assessed with unadjusted binary logistic regression. Associations between live birth and type of donor, menopausal status of donor, total ischemic time, and rewarming ischemic time were evaluated with unadjusted binary logistic regression. The reason to use unadjusted binary logistic regression was the large number of potential confounders involved and the small, albeit encouraging, number of cases currently included in the registry. A *P*-value < 0.05 was selected as the threshold of statistical significance.

## Results

### Uterus transplantation activity and geographical spread

Of the 91 UTx procedures analyzed, the majority (73.6%) were with uterine grafts from LD, with fewer (26.4%) being with uteri from deceased donors (DD). The geographical spread of the 91 UTx procedures by continent included 52 transplants in Europe (39LD/13DD; 75% LD UTx), 26 in Asia (22LD/4DD; 84.6% LD UTx), 4 in South America (2LD/2DD; 50% LD UTx), 3 in Australia (3LD/0DD; 100% LD UTx), and 6 in North America (1LD/5DD; 20% LD UTx). The median (ranges) follow-up time of patients with ongoing grafts was 15 (2–109) months, and for patients with removed grafts, the median (ranges) follow-up time was 30 (3–106) months.

### Donor and recipient characteristics

The demographic characteristics of the donors and recipients, including obstetrical data of donors, are presented in Table 1. The mean age of LDs was significantly higher than for DDs (48.7 years vs. 33.3 years; *P* < 0.001). The mean age for recipients was 29.5 years, with the youngest and oldest being 21 years and 42 years, respectively. Recipients had mean and median BMI values within the normal range, though individual values extended from 16.9 to 31.8 kg/m^2^. Mean and median BMI were comparable between DDs (24.3/23.9 kg/m^2^) and LDs (25.3/25.1 kg/m^2^), though LD values were marginally above the upper limit of normal (24.9 kg/m^2^). Of note, LDs had significantly more previous births than DDs (mean: 2.7 vs. 1.3; *P* < 0.001), while miscarriages and ectopic pregnancies were less frequent in both groups. Comorbidities were present in 16.5%, 26.9%, and 12.5% of recipients, LDs, and DDs, respectively. The incidence of different comorbidities and combinations of these in LDs, DDs and recipients are specified in Supplementary Table S1.

**Table 1.** Characteristics of donors and recipients.

Individual	Characteristic	*N*	Mean (SD)	Median (range)	Unknown (n)
Live donor	Age (year)	67	48.7 (7.6)	50.0 (30–62)	0
	BMI (kg/m^2^)	65	25.3 (4.4)	25.1 (17.7–36.5)	3
	Previous births	67	2.7 (1.1)	2.5 (1–6)	0
	Previous miscarriages	65	0.1 (0.4)	0 (0–2)	2
	Previous ectopic	65	0.0 (0.1)	0 (0–1)	1
Deceased donor	Age (year)	24	33.3 (11.7)	28 (18–57)	0
	BMI (kg/m^2^)	20	24.3 (3.2)	23.9 (19.9–32.0)	4
	Previous births	21	1.3 (1.3)	1.0 (0–4)	3
	Previous miscarriages	16	0.3 (0.6)	0.0 (0–2)	8
	Previous ectopic	16	0 (0)	0 (0)	8
Recipient	Age (year)	91	29.5 (4.6)	30.0 (21–42)	0
	BMI (kg/m^2^)	90	23.0 (3.7)	22.5 (16.9–31.8)	1

*Notes:* Previous births = vaginal + caesarian; when only one was known, it was assumed the other was zero.

Where number of previous births was recorded as >5, >5 was replaced with 6.

Previous smoking was significantly (*P* < 0.001) more common among DDs (7/17; 41.2% [7 unknown]) than among LDs (12/67; 17.9%), with an even lower rate (7/91; 7.7%) among recipients. All LDs except two were directed donors. Most of the LDs were mothers (43/67; 64.2%), with the rest being either a different relative (18/67; 26.9%) or someone else (6/67; 8.9%). Menopausal status was known for all 67 LDs and 22 of 24 DDs; among these, 38.8% (26/67) of LDs and 4.5% (1/22) of DDs were postmenopausal.

The vast majority (80/91; 87.9%) of the 91 recipients had the Mayer-Rokitansky-Küster-Hauser syndrome (MRKHs), with other AUFI causes being hysterectomy due to peripartum hemorrhage (5/91; 5.5%), hysterectomy for cervical cancer (3/91; 3.3%), or another reason (3/91; 3.3%). Of the 80 MRKHs patients, a majority had bilateral, normally positioned kidneys (71/80; 88.8%) and a vaginal epithelium-lined neovagina (non-dilated, self-dilated or Vechietti dilated; 66/79; 83.5% [1 unknown]).

Infectious risk constellations between donor and recipient were assessed through serology testing for CMV, EBV, and Toxoplasma. The results of testing for presence of hrHPV in vaginal swabs were also provided. Details of results are given in Supplementary Table S2. High risk donor + and recipient- constellations with more than 10% of donor/recipient high risk combinations were identified for CMV in LD UTx procedures (15.2%), for EBV in DD UTx procedures (22.2%), and for Toxoplasma in LD UTx procedures (10.6%). The rate of hrHPV positivity was low, being present in only 3.4% of LDs.

### Surgeries

**Figure 2.**
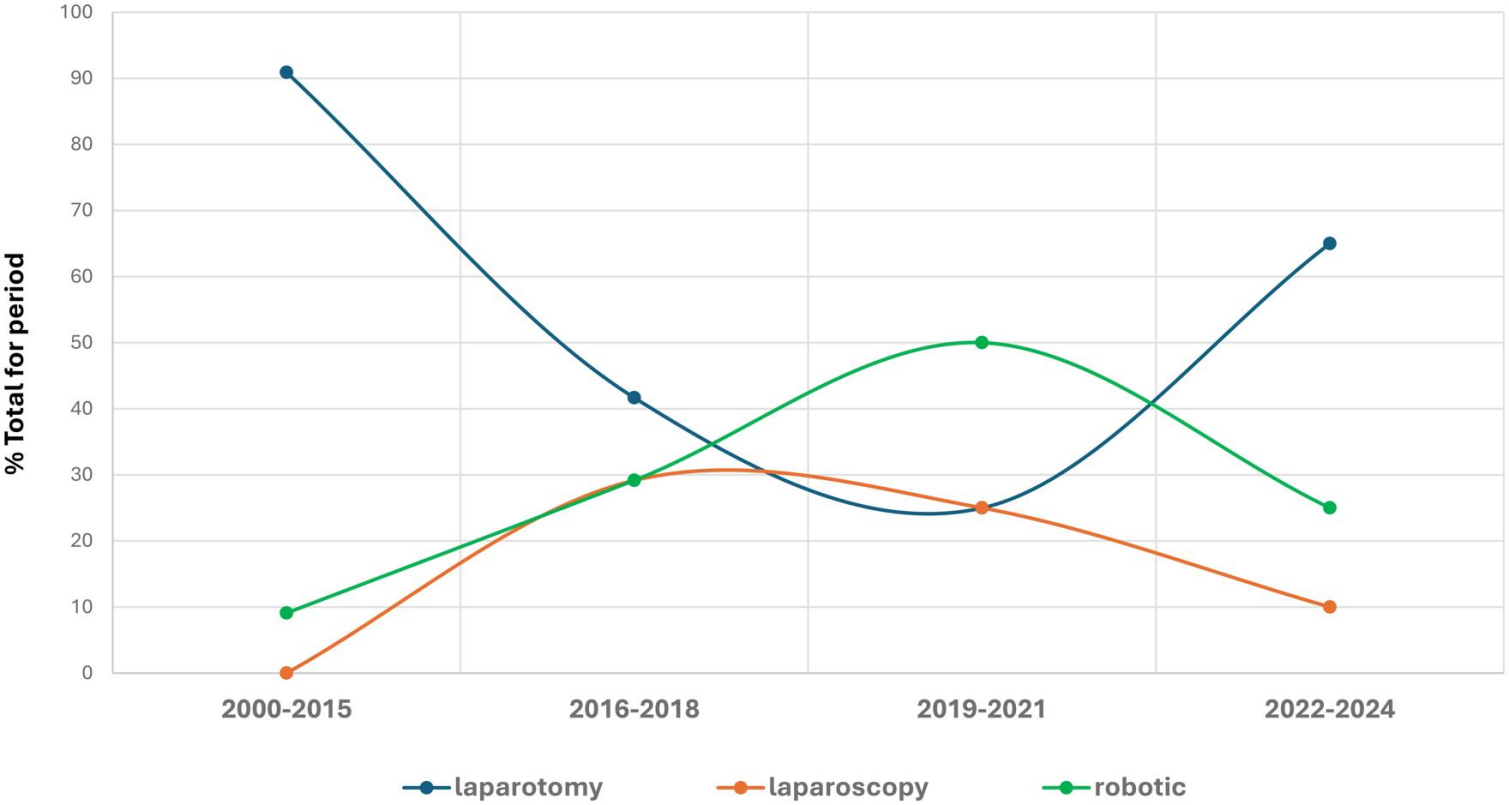
**Trends in surgical techniques used over time for live donor (LD) hysterectomies**.

Minimal invasive surgery (MIS) was used in 31 of the 67 (46.3%) LD hysterectomies (robotics used in 19 [28.4%] and laparoscopy in 12 [17.9%]). The time-dependent trends of the three different surgical techniques (laparotomy, laparoscopy-assisted, robotic-assisted) for LD hysterectomy are shown in Fig. 2. Of the 91 transplantations performed, MIS (robotics) was used in two recipient cases, while the remaining 89 were performed by laparotomy.

**Figure 3.**
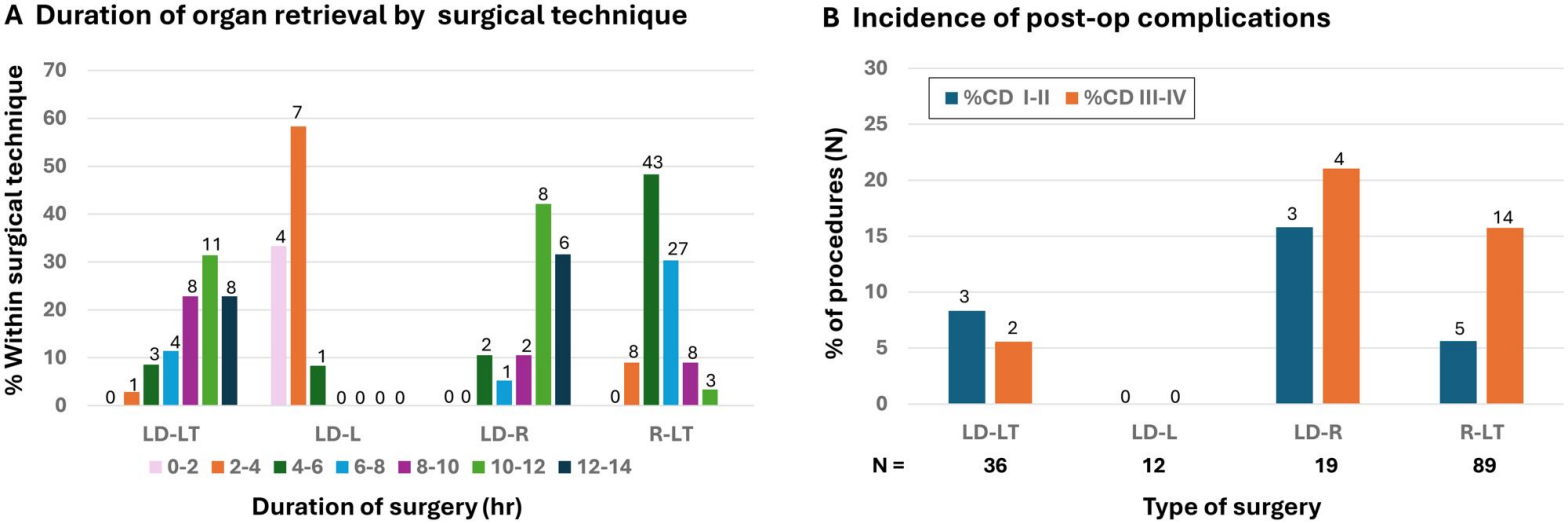
**Distributions of surgery durations and postoperative complications across surgical procedures.** (**A**) Durations of surgeries by three different techniques in live donor (LD) hysterectomies and in recipient surgery by laparotomy. Value for the duration of one LD-LT is missing. (**B**) Distribution of postoperative complications of Clavien-Dindo ≥ II within 90 days after surgery of all LD procedures and in all transplantation procedures by laparotomy. LD-L: live donor laparoscopy; LD-LT: live donor laparotomy; LD-R: live donor robotics; R-LT: recipient laparotomy.

The distributions of durations of surgeries in LDs and recipients are summarized in Fig. 3A. Durations over 10 h were in seen in 19/35 (54.3%; 1 unknown) of LD donor hysterectomies by laparotomy and in 14/19 (73.7%) of LD donor hysterectomies by robotics. Transplantations by laparotomy in the recipient lasted 4–6 and 6–8 h in 43/89 (48.3%) and 27/89 (30.3%), respectively. The two transplantations by robotics lasted 8–10 h.

The incidences of post-operative complications in the different types of surgeries are shown in Fig. 3B. The rate of CD III complications in LD hysterectomy by laparotomy, in LD hysterectomy by robotics, and in transplantation by laparotomy were 2/36 (5.6%), 4/19 (21.1%), and 14/89 (15.7%), respectively. In the two robotic transplantations, one was without postoperative complication, and one was with a CD III complication, which was graft removal. No CD IV complication was observed in any group.

Bilateral donor oophorectomy was performed in 16 of the 67 (23.9%) LDs, with 8 being from premenopausal women (age range 47–53 years), while unilateral oophorectomy was performed in 6 of 67 (9.0%), with 5 being premenopausal (age range 46–53 years). A blood loss of ≤ 600 ml occurred in 76.4% of recipient surgeries (2 unknown), in 100% of MIS LD donor hysterectomies, and in 79.4% of LD laparotomy procedures. Blood loss > 1000 ml occurred in 3/67 (4.5%) of LD donor hysterectomies and in 8/91 (8.8%) of recipient surgeries.

Durations of total ischemia times and rewarming ischemia times by surgical procedure were compared (Supplementary Table S3). Total ischemia duration was shortest for LD procedures by laparoscopy (LD-L: 1.0 ± 0.8 h, mean ± SD), with progressively increasing durations for those by robotics (LD-R: 3.0 ± 1.2 h), laparotomy (LD-LT: 3.2 ± 1.1 h) and DD-procedures (6.4 ± 3.2 h). The mean rewarming ischemia time in the recipients across the four different organ procurement settings were between 0.7 and 1.5 h.

Figure 1 shows graft survival (up to 12 months) for LD and DD transplants separately for all years studied (2000–2024) as well as when combined for the subperiods 2000–2015, 2016–2018, 2019–2021, and 2022–2024. Graft survival to 12 months, across the entire study period was 79.2% for DD UTx procedures and 73.1% for LD UTx procedures. When combining both donor types, overall graft survival was 74.7%, with survival rates of 75.0%, 78.1%, 60.0%, and 75.0% for years 2000–2015, 2016–2018, 2019–2021, and 2022–2024, respectively. Graft survival during the first 12 months was more likely if the uterus came from a premenopausal donor than from a postmenopausal donor, 85.5% vs 48.1% (OR 6.34, 95% CI [2.26–17.84], *P* < 0.001). None of the other studied variables were associated with graft survival. A total of 24 uteri were removed during the initial 12 months, and the most common causes of graft failure were thrombosis (2/10 [20%] for premenopausal grafts and 7/14 [50%] for postmenopausal grafts) and uterine hypoperfusion (4/10 [40%] for premenopausal grafts and 3/14 [21%] in postmenopausal grafts).

### Immunosuppression and rejections

Induction immunosuppression (IS) was used in the vast majority (88/91; 96.7%) of UTx procedures, with type of induction protocol and prevalence of usage being: thymoglobulin/anti-thymocyte globulin + steroids in 45/88 (51.1%), basiliximab + steroids in 19/88 (21.6%), only thymoglobulin/anti-thymocyte globulin in 8/88 (9.1%), only basiliximab in 7/88 (8.0%), steroids and “other” in 5/88 (5.7%), thymoglobulin/anti-thymocyte globulin + ‘other’ in 2/88 (2.3%), basiliximab + thymoglobulin/anti-thymocyte globulin in 1/88 (1.1%), and only “other” in 1/88 (1.1%).

Maintenance IS after transplantation was utilized in 90 of the recipients, as long-term IS was not provided to one recipient who had a transplant from her monozygotic twin sister. The maintenance IS included a calcineurin inhibitor (CNI) + mycophenolate mofetil (MMF) + steroids in 36/90 (40%), CNI + MMF in 25/90 (27.8%), CNI + azathioprine (AZA)+steroids in 13/90 (14.4%), CNI+ AZA in 12/90 (13.3%), CNI monotherapy in 2/90 (2.2%), and not specified in 2/90 (2.2%) cases. In the 88 cases with specified use of CNI, tacrolimus was the CNI in 87/88 (98.9%) and cyclosporine in 1/88 (1.1%). Complications related to IS were observed in 23 of 90 patients (25.6%) as follows: creatinine levels above 100 µmol/l for > 1 month in 6/90 (6.7%), hematological cytopenia in 4/90 (4.4%), diabetes in 3/90 (3.3%), hypertension in 2/90 (2.2%), opportunistic infection in 2/90 (2.2%), diabetes + hypertension in 1/90 (1.1%), hypertension + opportunistic infection + hematological cytopenia in 1/90 (1.1%), malignancy in 1/90 (1.1%), and other in 3/90 (3.3%).

Early rejection episode(s), i.e. those occurring in months 1–5 post-UTx, were identified in 31 of the 71 recipients (43.7%) who retained their grafts for at least 5 months. Most (22/31; 71.0%) of these recipients with early rejection(s) had only one rejection episode during this period, while only two had four episodes, which was the maximum number of early rejection episodes observed. For each recipient with a rejection, the severity of the rejection was determined by the highest grade (mild, moderate, or severe) observed across the period. Of the 31 patients with early rejections, they progressed to no higher than mild rejection in 20 patients, no higher than moderate rejection in eight patients, and to severe rejection in three patients. Treatment for the highest grade of rejection observed in each of the 31 patients included: only steroids (n = 20), steroids + CNI increase (n = 3), CNI increase (n = 1), steroids + AZA (n = 2), steroids + CNI increase + AZA (n = 2), steroids + CNI increase +other (n = 1), thymoglobulin/anti-thymocyte globulin + steroids + CNI increase + other (n = 1), and other treatment (n = 1). The three severe rejections during the early period were treated with thymoglobulin/anti-thymocyte globulin + steroids + CNI increase + other medication in one case, steroids + CNI increase + AZA in one case, and steroids + CNI increase in one case.

Later rejection episode(s) i.e. those occurring in months 6–10 post-UTx, occurred in 19 of the 68 (27.9%) recipients who retained their grafts to at least 10 months post-UTx. Most patients with late rejections (11/19; 68.7%) had only one rejection episode, but three patients had ≥ 5 rejection episodes during this 5 month-period. Of the 19 patients, late rejection progressed to no higher than mild grade in 12, no higher than moderate grade in four, and to severe grade in three. Treatment for the highest grade of rejection observed in the 19 patients included: only steroids (n = 14), steroids + CNI dose increase (n = 1), steroids + CNI increase + AZA (n = 1), steroids + thymoglobulin/anti-thymocyte globulin + AZA (n = 1), thymoglobulin/anti-thymocyte globulin + steroids (n = 1), and only thymoglobulin/anti-thymocyte globulin (n = 1). The three severe rejections during the late period were treated with thymoglobulin/anti-thymocyte globulin + steroids + AZA in one case, steroids + CNI increase in one case, and thymoglobulin/anti-thymocyte globulin + steroids in one case.

All rejection episodes during the first 10 months were reversible with antirejection therapy.

### Reproductive outcomes

A total of 44 live births were registered until the end of December 2024 from 36 of the 76 transplants (58 LD UTx, 18 DD UTx) performed before July 2023, with 36 children being the first child from a specific recipient and 8 the second child, yielding an overall live birth rate (at least one live birth) of 36/76 (47.4%).

Among the 58 recipients with uteri from LDs, 28 women had live births: 20 delivered one child each, and 8 delivered two children. Among the 18 recipients with uteri from DDs, 8 women had live births, delivering one child each. The overall live birth rates (at least one birth) were 48.3% and 44.4% for LD and DD procedures, respectively.

Among the 36 recipients with live births, 35 underwent transplantation by laparotomy, while one received a robotic transplantation (from a robotic LD hysterectomy). The types of donor hysterectomy surgery in the 28 women giving birth after LD UTx procedures were laparotomy in 16 (57.1%), robotics in 11 (39.3%), and laparoscopy in 1 (3.6%).

The overall live birth rates (at least one birth) for the LD procedures after LD hysterectomy by laparotomy, robotics, and laparoscopic surgery were 16/29 (55.2%), 11/17 (64.7%), and 1/12 (8.3%), respectively.

The origin of sperm for the 44 live births was the recipient’s partner for 42 children, and a sperm donor for two children, both of whom were delivered from the same recipient. All recipients used their own oocytes for the live births; in 14, the oocytes were retrieved after UTx. Pre-implantation genetic testing for aneuploidy (PGT-A) was used for 7/44 (15.9%) embryos that led to a live birth. The median age of the mothers at transplantation who used PGT-A was 31 years (range 21–36 years).

The numbers of ETs to achieve the 44 live births were 1 ET for 18 live births (40.9%), 2 or 3 ETs for 14 live births (31.8%), 4–6 ETs for 6 live births (13.6%), 7 or 8 ETs for 3 live births (6.8%), and ≥ 14 ETs for the remaining 3 live births (6.8%). The stage and number of embryos resulting in the 44 live births were single blastocyst (n = 38), single Day-2 (n = 4), single Day-3 (n = 1), and three embryos transferred (stages not specified; n = 1). When the number of ETs was truncated to 15, the live birth rate per ET was 30.3%, with a mean of 3.30 ETs (SD 3.61; range 1–15) required to achieve a live birth.

Maintenance IS after transplantation was utilized in 43 of the 44 pregnancies with live births (maintenance IS was not needed for the one recipient who had a transplant from her monozygotic twin sister). The most common regimen used was CNI+ AZA + steroids (in 29/43 [67.4%]), followed by CNI+ AZA (in 5/43 [11.6%]), CNI + steroids (in 4/43 [9.3%]) and CNI monotherapy (in 2/43 [4.7%]). Tacrolimus was the CNI used in all 43 cases. Rejections occurred in five pregnancies that led to live birth and were detected during weeks 1–12 in two of them and weeks 13–27 in three. In all five of these pregnancies, CNI+ AZA + steroids was the maintenance IS used. Treatments of the rejection episodes were increased steroid dose in four of the pregnancies and increase in steroids + CNI in one.

Delivery of the first child occurred in six women after one miscarriage and in one woman after 5 miscarriages. All births were singletons. The mean duration between UTx and the ET resulting in the first live birth was 21.3 months (SD 19.2; range 3.3–100.2 months). No statistically significant associations were found between total ischemic time, rewarming ischemia time, or donor type (LD or DD) and the chance of achieving at least one live birth. Recipients were significantly more likely to achieve live birth if the donor was premenopausal 57.1% (28/49) vs postmenopausal 28% (7/25) (OR 3.43, 95% CI [1.21–9.70]; *p* = 0.020). However, in subgroup analyses, excluding women with graft failure up to 12 months, there was no significant difference in live births between uterus transplants from premenopausal donors 28/40 (70%) and postmenopausal donors 7/12 (58%), (OR 1.67, 95% CI [0.44–6.32]; *P* = 0.45).

Any type of pregnancy complication occurred in 27/44 (61.4%) of pregnancies resulting in live birth, with the most common being preeclampsia (10/44; 22.7%), followed by gestational hypertension (5/44; 11.4%), and gestational diabetes (4/44; 9.1%). In 11 pregnancies, there was a combination of two or three complications (Table 2). None of the women with rejection episodes during pregnancy had infectious complications. Three of the eight second-child pregnancies had complications (Table 2), while all first pregnancies in these women were uncomplicated.

**Table 2.** Pregnancy complications among uterus transplantation recipients with pregnancies leading to live birth.

No. of recipients with complication (s)	GD	GH	PE	A/P	PREV	PROM	IUGR	ICP	R	H/B	SCH	INF	O	GW					Apgar			
4	−	−	**+**	−	−	−	−	−	−	−	−	−	−	35	31	32	37		10	9	8	10
2	−	−	−	−	−	**+**	−	−	−	−	−	−	−	35	36			10		10		
2	−	−	−	−	−	−	−	−	−	−	**+**	−	−	36	31			8		10		
2	−	−	−	−	−	−	−	−	−	−	−	**+**	−	33	35			7		10		
2	−	−	−	−	−	−	−	−	−	−	−	−	**+**	33	35*			10		10*		
1	**+**	−	**+**	−	−	−	−	−	−	−	−	**+**	−	31				6				
1	**+**	**+**	−	−	**+**	−	−	−	−	−	−	−	−	35				10				
1	**+**	**+**	−	−	−	−	−	−	−	−	−	−	**+**	36				9				
1	−	−	**+**	−	−	−	−	−	**+**	−	−	−	−	34					9			
1	−	**+**	−	**+**	**+**	−	−	−	−	−	−	−	−	37*					8*			
1	−	−	**+**	−	−	**+**	−	**+**	−	−	−	−	−	34					7			
1	−	**+**	−	−	−	**+**	−	−	**+**	−	−	−	−	33					9			
1	−	−	**+**	−	−	−	**+**	−	−	−	−	−	**+**	29					9			
1	−	−	−	−	−	−	**+**	−	−	**+**	−	**+**	−	33					9			
1	−	−	**+**	−	−	−	**+**	−	−	−	−	−	−	28					8			
1	−	−	**+**	−	**+**	−	−	−	−	−	−	−	−	34*					9*			
1	**+**	−	−	−	−	−	−	−	−	−	−	−	−	34					9			
1	−	**+**	−	−	−	−	−	−	−	−	−	−	−	36					10			
1	−	−	−	−	−	−	−	**+**	−	−	−	−	−	34					10			
1	−	−	−	−	−	−	−	−	**+**	−	−	−	−	33					10			
Total 27	4	5	7	1	3	3	3	2	3	1	1	3	3									

Data are also provided on gestational length (completed gestational) weeks and Apgar score at 5 min. A/P = placenta accreta/percreta; FGR = fetal growth restriction; GD = gestational diabetes; GH = gestational hypertension; GW = gestational week; H/B = hematoma/bleeding; ICP = intrahepatic cholestasis of pregnancy; INF = infection; O = other; PE = preeclampsia; PREV = placenta previa; PROM = premature rupture of membranes; R = rejection; SCH = subchorionic bleeding. * = Indicates a pregnancy for the second child.

In all live births, delivery was by cesarean section. Delivery was performed per protocol in 21 cases, but was indicated for preeclampsia in 8, bleeding in 3, premature rupture of membranes in 2, other uterus-related complications in 1, fetal growth restriction in 1, other complication of the child in 1, infection in 1, another complication in 5, and was not specified in 1.

The gestational age, calculated by completed gestational weeks at parturition, for the 44 live births was 34.5 ± 2.4 weeks (mean ± SD; range 28–38 weeks). Of the 44 children, 33 (75.0%) were born prematurely (<37 weeks of gestation), however none were extremely premature (<28 weeks of gestation). Twelve (36.4%) of the premature children were born per protocol. The overall birthweights among the 44 neonates were 2407 ± 95 g (mean ± SD; range 750—3660 g). Apgar scores of <7 were observed at 1, 5, and 10 min in 6, 1, and 0 neonates, respectively. Major postnatal complications occurred in 11 (25.0%) infants, with 9 having respiratory distress, 1 having cardiovascular disease, and 1 having another complication. No case of major malformation was reported.

### Hysterectomy and health status 3-months post uterine removal

At the time that data extraction ended, uterine removal had occurred in 65 of the 91 procedures, with 29/65 (44.6%) being after one (n = 21) or two (n = 8) live births. The reasons for uterine removal for these 29 recipients who had given birth were per protocol (10/29; 34.5% [all of the 8 recipients with two live births and 2 with one live birth]), patient′s request (7/29; 24.1%), side effects of IS (6; 20.7%), intrauterine infection (2; 6.9%), rejection (2; 6.9%), and one case each of unspecified and repeated miscarriages/multiple implantation failures.

The reasons for hysterectomy for the 36 recipients who did not accomplish live birth before uterine removal were linked to uterine hypoperfusion (12/36; 33.3%), thrombosis (9/36; 25.0%), repeated miscarriages/implantation failures (8/36; 22.2%), intrauterine infection (2/36; 5.6%), and one case each of rejection, patient’s request, PTLD, irreversible endometrial damage, and unspecified.

Across all cases involving uterine removal, all recipients, all LDs, and all children were alive at the 3-month follow-up.

## Discussion

Uterus transplantation has advanced from a pioneering experimental procedure into a clinical reality for women with AUFI. This second ISUTx registry report expands the dataset from the 45 UTx procedures across four continents in the first report (Brännström *et al.*, 2023a), to now encompass 91 procedures across five continents. The presented data provides a comprehensive global view of the UTx landscape, containing detailed data and trends on all relevant aspects of UTx and will thus serve as the compendium for quality assessment and process improvement utilized by both clinicians in the field and policymakers.

The number of UTx procedures and the number of active centers have doubled since the first report (Brännström *et al.*, 2023a), demonstrating increasing global application and interest. Notably, this registry update includes the results from 12 new centers, contributing 25 new cases to the registry overall. Notably, this report contains cases performed at one center in the United States, marking an encouraging step toward broader global participation in the registry. As a primary goal of the ISUTx is to compile UTx data from around the world in order to provide evidence-based practice recommendations to the global community, it is challenging to achieve this goal if a significant fraction of global activity is not captured in the registry. While no procedure in Africa has been entered into the registry, the ISUTx is not aware of any past or present UTx activity on that continent. In contrast, there are four centers (three in the USA and one in Australia) that have performed UTx but have not been reported to the ISUTx registry and thus could not be included in this report. While outcomes of many of these U.S. cases were recently published in a separate report (Johannesson *et al.*, 2022), the absence of these cases in the ISUTx registry poses challenges to global benchmarking efforts. While it is self-evident how missing large numbers of cases limits the robustness of conclusions about the global success of the field, it cannot be emphasized enough that all cases count, and the impact of missing any cases may be significant. Efforts thus remain underway to overcome any barriers to case-reporting to the ISUTx registry so that our community can provide truly comprehensive and accurate outcomes reporting and promote knowledge exchange that accelerates progress in the field.

Early milestones in the field of UTx demonstrated the surgical feasibility of the procedure using deceased donor (DD) as early as 2011 (Ozkan *et al.*, 2013) and then live donor (LD) in 2012 (Brännström *et al.*, 2014). The first live births following LD UTx and DD UTx occurred in 2014 in Sweden (Brännström *et al.*, 2015) and 2017 in Brazil (Ejzenberg *et al.*, 2019), respectively. Despite the early landmark achievements in DD UTx and the obvious lack of surgical risk when utilizing a DD, data from the current study show that LD UTx continues to predominate, accounting for 74% of all procedures. However, there has been a modest increase in the proportion of DD UTx, rising from 22% in the previous registry report (Brännström *et al.*, 2023a) to 26% in the present study. This slight upward trend may reflect increased confidence in DD protocols and evolving logistical capabilities in DD access. While the availability of DDs is expected to remain low in certain European countries, such as France (Dion *et al.*, 2022), other regions, including Australia, appear to offer more promising conditions for DD UTx, with a more favorable donor landscape (Pittman *et al.*, 2023).

In this report, we present comprehensive data on the demographics and clinical characteristics of both donors and recipients. Consistent with findings from the first registry report (Brännström *et al.*, 2023a), LDs were significantly older than DDs. The relatively advanced age of LDs, 64% of whom were the recipients’ mothers, is further underscored by the fact that 39% of LDs were postmenopausal at the time of uterus donation. Notably, all LDs had a history of childbirth, offering a form of functionality validation of uterine capability before transplantation. In contrast, this was not always the case among DDs, as nulliparity was observed in this group.

The presence of one or more comorbidities was more common among LDs (27%) compared to DDs (12%), a difference that is not unexpected given that the mean age of LDs was approximately 15 years higher than that of DDs. The most frequently observed comorbidity was hypertension, which, when adequately controlled with medical therapy from an early stage, is not expected to affect surgical outcomes negatively. Previous smoking was reported in 41% of DDs compared with 18% of LDs. However, the potential negative impact of the higher smoking prevalence among DDs may be mitigated by the considerably younger age of DDs as compared to LDs.

Mayer-Rokitansky-Küster-Hauser syndrome remains the predominant indication for UTx, accounting for 88% of recipients. Despite remaining high, this proportion is lower than the 98% of MRKH cases reported in the previous registry report (Brännström *et al.*, 2023a). The current dataset includes 11 women with non-MRKH causes of AUFI, all of whom had undergone hysterectomy due to benign or malignant conditions. Collectively, these findings indicate a broadening of the underlying causes of AUFI among UTx recipients, an expansion that is likely to continue in the future. A high proportion (83%) of recipients with MRKHs had a neovagina lined with vaginal epithelium. This anatomical feature may offer a protective advantage against ascending infections in immunosuppressed recipients. It could reduce the risk of miscarriage, compared to neovaginas constructed with intestinal, peritoneal, or skin grafts. Supporting this notion, one transplanted patient with a McIndoe neovagina lined with a split-thickness skin graft experienced multiple miscarriages and no live birth despite 14 ETs (Brännström *et al.*, 2022).

Viral serology of donor EBV-positive/recipient EBV-negative mismatch was seen in 22% of DD UTx procedures. This type of serology mismatch is known to increase the risk of EBV infection and PTLD (Rouce *et al.*, 2014). Indeed, one DD recipient of the registry developed PTLD, leading to discontinuation of IS and graft removal within the first-year post-transplantation. Among LD UTx procedures, donor CMV-positive/recipient CMV-negative mismatch was observed in 15%. While there are limited reports of successful transplantation of high-risk CMV-mismatched uterus transplants (Rosenzweig *et al.*, 2021), mismatch confers a high risk of primary CMV infection in the recipient post-transplant, which in solid organ transplantation has been associated with increased morbidity and graft dysfunction (Banga *et al.* 2025; Perry *et al.*, 2025). Typically, antiviral prophylaxis is administered, but only for a limited period after transplantation. Most importantly, treatment for invasive CMV infection in pregnancy is significantly limited by a lack of pharmacologic agents with an established safety record in pregnant women. Fear of adverse consequences for both mother and baby likely explains the low prevalence of these high-risk uterus transplants.

Pertaining to types of surgery conducted, the data show that hysterectomies in LD UTx were initially performed almost exclusively via open laparotomy. However, consistent with broader advancements in gynecologic and transplantation surgery, MIS techniques have increasingly been introduced for the technically demanding and time-consuming LD donor hysterectomy. In our analysis of surgical trends over time, we found that the great majority of LD hysterectomies performed during the early phase (2000–2015) were conducted via laparotomy. Minimally invasive surgery approaches, predominantly robotic-assisted LD hysterectomy, became more prevalent during the 2016–2018 and 2019–2021 periods, surpassing laparotomy in frequency. Surprisingly, however, the most recent study period (2022–2024) saw a reversal in this trend, with approximately 65% of LD hysterectomies performed via open laparotomy. This recent shift, from robotic-assisted LD hysterectomy to laparotomy LD hysterectomy, is unexpected given the well-established benefits of robotic-assisted surgery seen in hysterectomy for gynecologic cancer, including shorter hospital stay, less bleeding and quicker return to normal daily activities (Eklind *et al.*, 2015). A likely explanation lies in the increase in new centers during 2022–2024, compared with 2019–2021, as it is common for a new center to begin with laparotomy, a technique familiar to both gynecologic and transplant surgeons, before transitioning to robotics as clinical experience accumulates. This stepwise surgical evolution has been thoroughly documented by the pioneering LD UTx center in Sweden (Brännström *et al.*, 2020) and by the largest UTx center in the USA (Jacques *et al.*, 2024). Additionally, limited access to robotic platforms due to their high cost represents a practical barrier, particularly for newer or resource-constrained centers, which may further explain the resurgence of laparotomy in recent years. Notably, two of the recipient transplantation procedures were performed using robotic-assisted surgery, while the remainder were carried out via conventional open laparotomy.

Operative times for LD hysterectomy were prolonged beyond typical durations for both laparotomy and robotic surgery, with the majority exceeding 10 hr. In contrast, recipient procedures performed via laparotomy generally lasted 2–6 h, approximately half the duration of LD hysterectomies. Continued refinement of surgical techniques and accumulation of experience are expected to substantially reduce operative times, particularly for the technically demanding LD hysterectomy.

Postoperative complications within 90 days of UTx surgery were recorded and classified according to Clavien–Dindo (CD) grades. No CD grade IV complications occurred in any group; however, a notable proportion (21%) of CD grade III complications was observed following robotic-assisted LD hysterectomy. Complications of this grade require surgical, endoscopic, or radiological intervention. However, the registry does not contain any details on types of CD grade III complications, but a recent review pointed out that postoperative complications of the urinary tract were common among LDs with postoperative complications (Brännström *et al.*, 2023b). It is anticipated that complication rates following LD hysterectomy, especially with robotic surgery, will decline over time as experience and technical refinements accumulate. However, clearly an opportunity exists for the field to improve DD access, to avoid morbidity to LDs. The relatively high rate (16%) of CD grade III complications among recipients within 90 days following transplantation surgery was exclusively attributable to surgical interventions related to hysterectomy of failed grafts.

Graft survival rates up to 12 months, prior to any live birth, were approximately 75% overall and across most study periods, with a temporary decline to around 60% during 2019–2020 for reasons that remain unclear. These figures are comparable to those reported in the smaller U.S. cohort of 33 procedures, which demonstrated a graft survival rate of 74% (Johannesson *et al.*, 2022). Our data further show that most graft losses occur within the first 6 months and are primarily attributable to insufficient graft perfusion, typically resulting from initial hypoperfusion or thrombosis. In an analysis of early graft failures in the Swedish studies conducted between 2012 and 2017, early indicators of graft loss, which developed within a few months, were identified (Karlsson *et al.*, 2022). These included low initial central uterine blood flow, with color Doppler signals confined to the periphery of the uterus and absent toward the central cavity. The authors further speculated that several graft failures might have resulted from small uterine artery diameters in grafts from older LDs or from impaired venous outflow. In the present study, graft failure was more common in postmenopausal uterus grafts. Moreover, 67% of all graft failures (71% of the transplants with postmenopausal grafts and 60% of the transplants with premenopausal grafts) were due to either hypoperfusion or thrombosis. The association between menopausal status and graft failure will be important to follow up on in future studies with more data. Whether the risk of graft failure is increased in postmenopausal grafts due to insufficient graft perfusion is a hypothesis that needs to be further explored.

Immunosuppression management strategies included induction therapy in a great majority of cases, using a variety of induction agents. Tacrolimus remains the cornerstone of maintenance IS. The rates of acute rejection episodes were 44% and 28% during months 1–5 and 6–10 after transplantation, respectively. The corresponding rates for early and late rejection episodes in the previous report (Brännström *et al*., 2023a) were 33% and 11%. Rejection rates among registry cases were comparable to those reported in the US series, where 43% of the 23 patients that showed graft survival over for 12 months had one or more acute rejection episodes (Johannesson *et al.* 2022). All acute rejection episodes within the first 10 months post-UTx were successfully reversed, most commonly with corticosteroid therapy as an adjunct immunosuppressive measure. Notably, the data continue to show that some degree of rejection, also during pregnancy, does not necessarily preclude a successful pregnancy. However, close monitoring and timely intensity of IS has remained critical for favorable outcomes.

A strictly successful UTx procedure is defined by the delivery of a healthy child from the transplanted uterus, thereby demonstrating the graft’s capacity to support embryo implantation and sustain pregnancy to neonatal viability. The present study constitutes the most extensive compilation to date of pregnancies resulting in live births after UTx, comprising 44 live births from 36 mothers. In comparison, two previous reports each described 19 live births (Johannesson *et al.*, 2022; Brännström *et al.*, 2023a).

Live birth rates were comparable between LD and DD UTx procedures, demonstrating the effectiveness of both approaches. However, within the LD cohort, outcomes varied substantially depending on the surgical technique used for donor hysterectomy, with live birth rates of 8% after conventional laparoscopy, 55% after laparotomy, and 65% after robotic-assisted surgery. The reasons behind the lower success rates seen with conventional laparoscopic uterine retrieval remain unclear, but the current data indicate that this approach offers no meaningful advantage in terms of graft function or reproductive outcomes. In contrast, robotic-assisted laparoscopy has consistently demonstrated higher reliability and surgical precision. Taken together, these findings suggest that traditional laparoscopy is unlikely to remain a suitable technique for UTx retrieval, particularly in settings where robotic-assisted surgery is available.

Assisted reproduction using IVF was employed to achieve pregnancy in all cases. The live birth rate was 30.3% per ET, with blastocyst transfer being most common (86%). While this live birth rate/ET may seem on the low side, it is important to note that although the recipients were young (mean age, 29.5 years) and all used autologous oocytes, the age of the transferred uterus may have contributed to some of the failed implantations. Most of the recipients (73.6%) received uteri from LDs, and their mean age was 48.7 years. Thus, the somewhat low live birth rate per ET in this UTx cohort aligns with the well-established gradual decline in pregnancy rates with advancing uterine age, as demonstrated in an analysis of more than 30,000 oocyte donation cycles (Sebastián-León *et al.*, 2025) and the results of a systematic review and meta-analysis of more than 11,000 ETs involving euploid embryos (Vitagliano *et al.*, 2023).

The rate of pregnancy complications was notably high (61%), with hypertensive disorders of pregnancy (preeclampsia or gestational hypertension) occurring in 34% of pregnancies. Notably, the incidence of hypertensive disorders of pregnancy among heart and lung transplant recipients exceeds 55% (Tanaka *et al.*, 2025). Several additive factors may contribute to the high rate of hypertensive disorders of pregnancy after UTx, including IVF using fully allogeneic embryos, as in donor oocyte cycles (Keukens *et al.*, 2022), immunosuppressive treatment (Tanaka *et al.*, 2025), a relatively high prevalence of single-kidney recipients (Artan *et al.*, 2023), and the relatively high proportion of transplanted uteri originating from women of advanced age (Carr *et al.*, 2022).

The mean gestational length was 34.5 weeks, with 75% of infants born preterm. The high rate of preterm delivery was partly attributable to the study protocol, as approximately one-third of these deliveries were scheduled electively. This approach represented a balance between achieving adequate fetal maturation and minimizing potential UTx-related obstetric complications in late pregnancy. The relatively high rate of respiratory distress may also reflect this practice of prematurely planned deliveries, which would not align with current management strategies of UTx pregnancies favoring longer gestation when clinically feasible.

A limitation of this registry report is that there is some degree of underreporting. To our knowledge, there are at least four centers (three in the USA and one in Australia) that have not yet contributed their cases. A great majority of these cases, which have not been entered into the ISUTx registry, were presented in an article reporting the experience of the first 5 years of UTx in the USA (Johannesson *et al.*, 2022). This gap of underreporting into the ISUTx registry continues to pose challenges to global benchmarking. It underscores the necessity of mandating registry participation for future clinical studies and within national regulatory frameworks. Another limitation is that this report is mainly descriptive, and regression analyses were not adjusted due to the limited amount of data. Future studies will further explore associations between background factors, surgery and graft survival, and reproductive outcomes.

In conclusion, this second ISUTx registry report provides the most comprehensive global overview of UTx to date. Continued international collaboration with standardized reporting will be essential to further enhance live birth rates, maternal safety, and neonatal health as UTx continues its transition from pioneering innovation to established clinical practice.

## Supplementary Material

deag017_Supplementary_Table_S1

deag017_Supplementary_Table_S2

deag017_Supplementary_Table_S3

## Data Availability

The specific data underlying this article will be shared on reasonable request to the corresponding author.
