# Supplementary material for: Second report of registry of the International Society of Uterus Transplantation (ISUTx): international activities 2000–2024
Source: Hum Reprod. 2026 Feb 17;41(4):541–51. doi: 10.1093/humrep/deag017 (PMC13061116; doi:10.1093/humrep/deag017)
Supplement: deag017_Supplementary_Table_S1 [file deag017_supplementary_table_s1.pdf]

OXFORD
human reproduction

Human Reproduction, 2026, 1–1
https://doi.org/10.1093/humrep/deag017
Supplementary Data

Supplementary Table S1. Incidence of comorbidities among live donors, deceased donors, and recipients.

Live donor (n = 67)	Comorbidity	N	% of total
	Hypertension only	5	7.5
	+ Diabetes	1	1.5
	+ Thyroid disorder	1	1.5
	+ Thyroid disorder + hyperlipidemia	1	1.5
	Thyroid disorder only	2	3.0
	+ Other	1	1.5
	Diabetes only	1	1.5
	+ Hyperlipidemia	1	1.5
	Hyperlipidemia only	1	1.5
	Previous non-uterine intra-abdominal surgery only	1	1.5
	+ Hyperlipidemia	1	1.5
	Previous intra-uterine intra-abdominal surgery only	1	1.5
	Other only	1	1.5
	**Any**	**18**	**26.9**
**Deceased donor (n = 24)**	**Comorbidity**	**N**	**% of total**
	Neurological disorder only	1	4.2
	+ Previous non-uterine intra-abdominal surgery	1	4.2
	Previous non-uterine intra-abdominal surgery only	1	4.2
	**Any**	**3**	**12.5**
**Recipient (n = 91)**	**Comorbidity**	**N**	**% of total**
	Previous non-uterine intra-abdominal surgery only	7	7.7
	Neurological disorder only	2	2.2
	Hyperlipidemia only	1	1.1
	+ Asthma/lung disease	1	1.1
	Asthma lung disease only	1	1.1
	Other only	3	3.3
	**Any**	**15**	**16.5**
