# Supplementary material for: Second report of registry of the International Society of Uterus Transplantation (ISUTx): international activities 2000–2024
Source: Hum Reprod. 2026 Feb 17;41(4):541–51. doi: 10.1093/humrep/deag017 (PMC13061116; doi:10.1093/humrep/deag017)
Supplement: deag017_Supplementary_Table_S2 [file deag017_supplementary_table_s2.pdf]

OXFORD
human reproduction
Human Reproduction, 2026, 1–1
https://doi.org/10.1093/humrep/deag017
Supplementary Data

Supplementary Table S2. Serological/viral mismatches/matches between donors and recipients.

	CMV		EBV		Toxoplasma		hrHPV	
	**LD**	**DD**	**LD**	**DD**	**LD**	**DD**	**LD**	**DD**
**Total donor/recipient serological combinations known**	66	23	64	18	47	9	59	12
% **donor+/recipient- of total**	10 (15.2)	2 (8.7)	3 (4.7)	4 (22.2)	5 (10.6)	0 (0.0)	0 (0.0)	0 (0.0)
% **donor-/recipient+ of total**	7 (10.6)	5 (21.7)	2 (3.1)	2 (11.1)	0 (0.0)	0 (0.0)	2 (3.4)	0 (0.0)
% **donor-/recipient- of total**	25 (37.9)	5 (21.7)	18 (28.1)	0 (0.0)	33 (70.2)	8 (88.9)	57 (96.6)	12 (100.0)
% **donor+/recipient+ of total**	24 (36.3)	11 (47.9)	41 (64.1)	12 (66.7)	9 (19.2)	1 (11.1)	0 (0.0)	0 (0.0)

Numbers in parentheses are the percentages of the total donor/recipient serological combinations known.
Only cases in which a serology/hrHPV combination was known are included.
CMV = Cytomegalovirus; DD = Deceased donor; EBV = Epstein-Barr Virus; hrHPV = High risk Human Papilloma Virus; LD = Live donor.
