# Supplementary material for: Second report of registry of the International Society of Uterus Transplantation (ISUTx): international activities 2000–2024
Source: Hum Reprod. 2026 Feb 17;41(4):541–51. doi: 10.1093/humrep/deag017 (PMC13061116; doi:10.1093/humrep/deag017)
Supplement: deag017_Supplementary_Table_S3 [file deag017_supplementary_table_s3.pdf]

OXFORD
human reproduction
Human Reproduction, 2026, 1–1
https://doi.org/10.1093/humrep/deag017
Supplementary Data

Supplementary Table S3. The relationship between surgical approach and total ischemia and rewarm ischemia times.

**Total ischemia time (h)**	**DD**	**LD-LT**	**LD-L**	**LD-R**
N	24	35	12	18
Mean + STD	6.4 ± 3.2	3.2 ± 1.1	1.0 ± 0.8	3.0 ± 1.2
Median	6.0	2.9	0.8	2.3
Range	1.5–15.1	1.0–6.4	0.8–3.5	1.3–5.2
**Rewarm ischemia time (h)**	**DD**	**LD-LT**	**LD-L**	**LD-R**
N	21	35	12	18
Mean + STD	1.2 ± 0.5	1.5 ± 0.3	0.7 ± 0.5	1.4 ± 0.5
Median	1.2	1.4	0.6	1.5
Range	0.4–2.1	0.7–2.4	0.3–2.3	0.5–2.4

DD = deceased donor; LD-L = live donor with laparoscopy; LD-LT = live donor with laparotomy; LD-R = live donor with robotic-assisted laparoscopy.

© The Author(s) 2026. Published by Oxford University Press on behalf of European Society of Human Reproduction and Embryology.
This is an Open Access article distributed under the terms of the Creative Commons Attribution License (https://creativecommons.org/licenses/by/4.0/), which permits unrestricted reuse, distribution, and reproduction in any medium, provided the original work is properly cited.
Human Reproduction, 2026, 00, 1–14
https://doi.org/10.1093/humrep/deag017
Original Article
